# Generative AI adoption and its impact on teachers’ self-efficacy and instructional confidence in Ghana

**DOI:** 10.3389/frai.2026.1829056

**Published:** 2026-06-18

**Authors:** Emmanuel Nana Kwesi Ofori Darko, Zhanyong Qi, Zhiyuan Wang

**Affiliations:** Faculty of Education, Shaanxi Normal University, Xi'an, China

**Keywords:** generative Artificial Intelligence (GenAI), Ghanaian K-12 education, institutional support, instructional confidence, teacher self-efficacy

## Abstract

**Background:**

The rapid integration of Generative Artificial Intelligence (GenAI) into education presents unique opportunities and challenges, particularly in developing nations. While research has explored factors influencing GenAI adoption, its subsequent impact on teachers’ core professional beliefs remains under-investigated. This study addresses this gap by examining the relationship between GenAI usage and two critical psychological factors teacher self-efficacy (TSE) and instructional confidence among K-12 educators in Ghana. It further investigates the moderating role of institutional support and professional development.

**Methods:**

An explanatory sequential mixed-methods design was employed. The quantitative phase involved a survey of 512 K-12 teachers from diverse districts in Ghana, utilizing validated scales to measure GenAI use, TSE (Teachers’ Sense of Efficacy Scale), instructional confidence, and perceived institutional support. Data were analyzed using descriptive statistics, correlation, and hierarchical multiple regression. The qualitative phase consisted of semi-structured interviews with a purposive sample of 20 teachers to provide deeper context and explanation for the quantitative findings.

**Results:**

Quantitative analysis revealed a significant positive correlation between the frequency of GenAI use and both teacher self-efficacy (*r* = 0.42, *p* < 0.001) and instructional confidence (*r* = 0.48, *p* < 0.001). Hierarchical regression models indicated that using GenAI specifically for lesson planning and creating differentiated instructional materials were the strongest predictors of higher TSE. Furthermore, institutional support was found to be a significant moderator, amplifying the positive association between GenAI adoption and instructional confidence. Qualitative analysis corroborated these findings, yielding two primary themes: GenAI as an “Efficiency Engine,” which alleviates workload in resource-constrained settings, and GenAI as a “Creative Catalyst,” which enhances pedagogical innovation and design.

**Conclusion:**

The findings suggest that the adoption of GenAI can serve as a potent tool for enhancing teacher self-efficacy and instructional confidence within the Ghanaian educational context. By automating routine tasks and providing novel pedagogical resources, GenAI empowers educators to focus on higher-order instructional strategies, thereby fostering greater professional competence. This effect is significantly strengthened when coupled with robust institutional support frameworks. This study offers critical insights for policymakers, school administrators, and teacher training institutions in Ghana and similar developing contexts, highlighting a strategic pathway for leveraging technology to empower the teaching workforce.

## 1 Introduction

The emergence of Generative Artificial Intelligence (GenAI) marks a paradigm shift in technology, with significant effects on many sectors, including education (Lim, 2023). Tools like ChatGPT, Gemini, and Claude are no longer new but are becoming part of educators’ daily routines worldwide, fundamentally changing traditional teaching methods and educational settings (Chiu, 2023; Börekci and Uyangör, 2025). Teachers are increasingly using these technologies for various tasks, such as lesson planning, creating instructional content, developing assessments, and customizing instruction for diverse learners (Walter, 2024; Asrori and Setyaningsih, 2025). This rapid adoption creates both significant opportunities and serious challenges (Giannakos, 2024), raising concerns about academic honesty, the risk of weakening critical thinking, and the need for new institutional policies (Michel-Villarreal, 2023; Hughes, 2025). In Ghana, a nation striving to modernize its education system amid infrastructural and resource constraints, the advent of GenAI presents both possibilities and challenges (Huang and Yan, 2025). The Ghana Education Service (GES) has been promoting the integration of ICT, yet the digital divide between urban and rural schools remains a persistent challenge. For Ghanaian teachers, who often manage large class sizes with limited teaching materials, GenAI could be a powerful ally, offering a means to create customized content and reduce administrative burdens. However, issues of access, digital literacy, and the lack of localized policies could also exacerbate existing inequities (Sun et al., 2025). While the global discourse on GenAI in education has primarily centered on student use and academic policy (Chan, 2023), the impact on teachers themselves remains a critical yet underexplored area. The successful integration of any new technology into the classroom is heavily dependent on the beliefs and attitudes of the educators tasked with its implementation (Chiu, 2023). Among the most crucial of these beliefs is teacher self-efficacy (TSE), defined as a teacher’s judgment of their ability to achieve desired outcomes in student engagement and learning (Zee and Koomen, 2016). Decades of research have established TSE as a powerful predictor of teacher effectiveness, persistence, commitment, job satisfaction, and ultimately, student achievement (Leithwood, 2008; Caprara et al., 2006).

Closely related to TSE is the concept of instructional confidence, which refers to a teacher’s assurance of their ability to perform specific pedagogical tasks and strategies effectively. While TSE reflects a broader belief in one’s overall capability, instructional confidence is more task-specific and reflects a teacher’s perceived readiness to handle classroom complexities. The introduction of GenAI tools could likely influence both constructs. On one hand, these tools may empower teachers by offering instant access to resources, ideas, and support, thereby increasing their sense of competence (Sohail et al., 2025). On the other hand, the steep learning curve, ethical uncertainties, and infrastructural challenges in a context like Ghana could create new anxieties and weaken confidence (Wang et al., 2024). The existing literature on GenAI adoption in education has primarily focused on identifying the reasons for use, often using frameworks such as the Technology Acceptance Model (TAM) or the Unified Theory of Acceptance and Use of Technology (UTAUT) (Kong, 2024). These studies consistently highlight self-efficacy as a key factor predicting technology adoption (Hazzan-Bishara et al., 2025; Mirriahi et al., 2025; Darayseh and Mersin, 2025). However, this relationship could be reciprocal; adopting and successfully using new technology can serve as a mastery experience that boosts self-efficacy. This feedback loop from adoption to confidence remains underexplored, especially in the context of GenAI, particularly in African educational settings.

This study aims to fill a crucial gap in the literature. We go beyond asking what predicts adoption to explore how adoption affects teachers’ professional beliefs in Ghana. By examining the relationships among GenAI use, teacher self-efficacy, and instructional confidence, we aim to understand how this transformative technology influences the psychological landscape of teachers in a developing country (Seran et al., 2025). Specifically, the research is guided by the following question: What is the relationship between the frequency and type of GenAI use and Ghanaian teachers’ overall self-efficacy? How does adopting GenAI tools impact teachers’ instructional confidence in areas like lesson planning, differentiation, and assessment? What role do contextual factors, especially institutional support and access to professional development, play in moderating the relationship between GenAI use and teachers’ efficacy beliefs? What are Ghanaian teachers’ personal experiences and perceptions regarding how GenAI affects their professional skills, confidence, and sense of competence? Using a mixed-methods approach, this study offers a detailed, data-driven understanding of the benefits and challenges of GenAI for the teaching profession in Ghana. The findings have important implications for developing practical teacher training, creating supportive school policies, and ultimately harnessing AI to empower, rather than overwhelm, educators in Ghana and similar settings.

## 2 Literature review

### 2.1 Generative AI in the educational landscape

The proliferation of GenAI tools has disrupted academic institutions worldwide, presenting transformative challenges and opportunities for decision-makers (Hughes, 2025). The potential benefits are extensive, including personalized learning support, assistance with writing and brainstorming, and improved research capabilities (Chan, 2023). For teachers, GenAI can serve as a powerful assistant, helping to generate lesson plans, create varied instructional materials, and design formative assessments, thereby boosting productivity and efficiency (Walter, 2024; Asrori and Setyaningsih, 2025; Sohail et al., 2025). This integration can lead to greater confidence and better strategic decision-making in instructional planning (Walter, 2024). However, this technological integration also presents challenges (Tongchai and Malakul, 2025). Concerns include the accuracy of AI-generated information, data privacy, biases in training data, and the risk of over-reliance, which can reduce students’ critical thinking skills (Chan, 2023). Some describe GenAI as a paradoxical force in education, both a ‘friend’ and a ‘foe’ (Lim, 2023). The adoption process is complex and influenced by factors at the individual, technological, and social levels (Russo, 2024). In developing regions like Ghana, these issues are worsened by digital inequality, infrastructure shortages, and the need for culturally relevant AI applications Huang and Yan (2025). Developing institutional policies and guidelines is essential for navigating this complex landscape (Jin et al., 2024); though, many education systems remain in the early stages of developing a clear response. Some GenAI represents a paradoxical influence in education serving as both a ‘friend’ and a ‘foe’ (Zhou and Li, 2026). Its adoption is complex, influenced by factors at the individual, technological, and social levels (Russo, 2024). In developing regions like Ghana, these challenges are intensified by digital inequality, infrastructure shortages, and the need for culturally relevant AI solutions. Establishing institutional policies and guidelines is crucial for managing this complex landscape (Jin et al., 2024), although many education systems are still in the early stages of formulating a clear response.

### 2.2 Teacher self-efficacy and its sources

Teacher self-efficacy (TSE) is a cornerstone of educational psychology, referring to a teacher’s belief in their own ability to organize and execute the actions required to accomplish a specific teaching task in a particular context (Klassen, 2014). Rooted in Bandura’s Social Cognitive Theory, TSE is a powerful determinant of teacher motivation, effort, and resilience (Lee and Stankov, 2018). It is commonly conceptualized across three domains: efficacy of instructional strategies, efficacy of classroom management, and efficacy of student engagement (Klassen et al., 2010). A strong sense of efficacy is linked to a host of positive outcomes, including greater job satisfaction, lower emotional exhaustion and burnout, and higher commitment to the profession (Skaalvik, 2014; Skaalvik, 2009; Schwarzer and Hallum, 2008). Crucially, higher TSE is also associated with improved instructional quality and student achievement (Holzberger et al., 2013; Caprara et al., 2006).

According to Bandura, self-efficacy beliefs develop through four main sources: mastery experiences (success in past performances), vicarious experiences (watching others succeed), social persuasion (encouragement from others), and physiological and emotional responses to tasks. Among these, mastery experiences are seen as the most impactful source (Πούλου, 2007). This theoretical foundation is central to the current study’s hypothesis: if GenAI adoption helps create successful teaching moments, such as designing an engaging lesson plan or providing a perfectly leveled text for a struggling reader, it can act as a powerful source of mastery experiences, thus boosting TSE. This is especially important for developing 21st-century teaching skills, where integrating technology is essential (Martínez, 2022). This is especially important for developing 21st-century teaching skills, in which integrating technology is essential (Martínez, 2022).

### 2.3 The role of self-efficacy in technology adoption

The relationship between self-efficacy and technology has been widely examined using adoption models such as TAM and UTAUT. These frameworks suggest that an individual’s belief in their ability to use a technology (i.e., self-efficacy) is either directly or indirectly linked to their intention to use it and their actual usage (McIntyre, 2024). Recent research on GenAI confirms this link within educational settings. For instance, studies indicate that teachers’ self-efficacy strongly predicts their intention to adopt GenAI (Darayseh and Mersin, 2025; Sun et al., 2026). Additionally, self-efficacy affects teachers’ perceptions of GenAI’s usefulness and ease of use, which in turn influence their willingness to adopt the technology (Hazzan-Bishara et al., 2025; Börekci and Uyangör, 2025). This trend is observed in both in-service (Elstad and Eriksen, 2024) and pre-service teachers (Li et al., 2026), as well as students (Mirriahi et al., 2025; Ursavaş et al., 2025). While this research is helpful, it mainly treats self-efficacy as a factor that leads to adoption. The current study shifts this view to explore how adoption, once it occurs, might also affect and enhance self-efficacy (Seran et al., 2025).

### 2.4 Instructional confidence and contextual supports

Instructional confidence, while closely related to the broader construct of TSE, is more task-specific. It reflects a teacher’s confidence in their ability to implement specific instructional practices successfully. The adoption of GenAI could enhance confidence in specific domains, such as creating personalized learning materials or managing administrative tasks (Butler-Ulrich et al., 2024). However, this potential is not realized in a vacuum. The educational environment plays a critical role. Institutional support, including professional development, access to resources, and clear policies, is a crucial factor. Studies have shown that institutional support has a direct and indirect influence on teachers’ intention to use AI (Hazzan-Bishara et al., 2025). Conversely, a lack of management support or discussion with colleagues can hinder the perceived utility of AI tools (Elstad and Eriksen, 2024). This support is also vital for teachers’ general wellbeing in technology-enhanced environments (Han, 2023). Leadership that fosters a clear vision and supports teacher development can mediate positive outcomes (Ahn, 2023). Therefore, this study conceptualizes institutional support as a key moderator that may amplify the positive effects of GenAI adoption on teachers’ confidence, a factor of particular importance in Ghana’s resource-variable context (McIntyre, 2024). Studies research has shown that institutional support directly and indirectly influences teachers’ intention to use AI (Hazzan-Bishara et al., 2025). Conversely, a lack of management support or discussion with colleagues can hinder the perceived utility of AI tools (Elstad and Eriksen, 2024). This support is also vital to teachers’ overall wellbeing in technology-enhanced environments (Shi et al., 2026). Leadership that fosters a clear vision and supports teacher development can mediate positive outcomes (Leithwood and Jantzi, 2008). Therefore, this study conceptualizes institutional support as a key moderator that may amplify the positive effects of GenAI adoption on teachers’ confidence, a factor of particular importance in Ghana’s resource-variable context (Tongchai and Malakul, 2025).

## 3 Methods

### 3.1 Study setting

This study was conducted in Ghana, a West African nation experiencing rapid changes in its educational system. The Ghanaian education system follows a K-12 structure, consisting of 6 years of primary school, 3 years of Junior High School (JHS), and 3 years of Senior High School (SHS). The curriculum is centrally overseen by the Ghana Education Service (GES) and developed by the National Council for Curriculum and Assessment (NaCCA), with recent focus on standards-based learning and the promotion of STEM and digital literacy. Although Ghana has made significant progress in increasing access to education, the use of technology in classrooms varies. Urban and private schools in areas like Greater Accra and Ashanti usually have better access to digital tools, including computers and internet, compared to rural and public schools in regions such as the Northern or Upper West. This digital gap creates a diverse environment for the adoption of technology. The government and various NGOs have launched initiatives to enhance digital literacy among teachers and students, creating an ideal context for exploring the adoption of emerging technologies, such as Generative AI. This environment presents a unique opportunity to examine how teachers in a developing economy, with varying levels of institutional support and resources, utilize GenAI and how this affects their professional confidence and effectiveness.

### 3.2 Research design

To provide a comprehensive understanding of the complex relationship between GenAI adoption and teacher beliefs, this study employed an explanatory sequential mixed-methods design. This two-phase approach started with the collection and analysis of quantitative survey data to identify statistical relationships and patterns among key variables. The second phase involved qualitative semi-structured interviews, designed to elaborate on and explain the quantitative results by exploring participants lived experiences and perspectives in greater depth. This design enables statistical generalization from the quantitative phase while offering rich, contextualized insights from the qualitative phase, presenting a nuanced view of the promises and risks of GenAI adoption (Sohail et al., 2025). This design enables statistical generalization from the quantitative phase while providing rich, contextualized insights from the qualitative phase, offering a nuanced view of the promises and risks of GenAI adoption (Sohail2025).

### 3.3 Participants and procedure

A total of 512 practicing K-12 teachers from Ghana participated in the survey. Participants were recruited through partnerships with District Education Offices, the Ghana National Association of Teachers (GNAT), and social media groups for Ghanaian educators. This approach aimed to ensure a diverse sample across various geographical regions, including urban, peri-urban, and rural areas, as well as different school types, such as public and private. To qualify, participants had to be currently employed K-12 teachers in Ghana and have used at least one GenAI tool (ChatGPT, Gemini, Copilot) for professional purposes. After giving informed consent via an online form, participants completed an online questionnaire in English, which took approximately 15–20 min. For the qualitative portion, respondents interested in follow-up interviews were purposively sampled, selecting 20 teachers to ensure maximum variation based on teaching experience (novice, mid-career, veteran), grade level (primary, JHS, SHS), school location (urban vs. rural), and self-reported frequency of GenAI use (low, medium, high). This strategy aimed to gather a rich diversity of perspectives to complement the quantitative data. The semi-structured interviews were conducted via phone or WhatsApp video call, lasted between 45 and 60 min, and were audio-recorded and transcribed verbatim for analysis.

### 3.4 Instruments

The quantitative survey consisted of four main sections: Demographics and Contextual Information, where participants provided details about their years of teaching experience, primary subject area, grade level, school type (public/private), and region. The GenAI Adoption Scale, developed for this study, included six items to measure how often teachers use GenAI for specific instructional tasks, drawing on the current literature on teachers’ applications of GenAI (Chiu, 2023). Participants rated their frequency of using GenAI for (a) lesson planning, (b) creating instructional materials, (c) differentiating instruction, (d) generating assessments, (e) communicating with parents/students, and (f) professional administrative tasks. Responses were given on a 5-point Likert scale from 1 (Never) to 5 (Very Frequently). An overall GenAI Adoption score was then calculated by averaging responses. The Teachers’ Sense of Efficacy Scale (TSES—Short Form), a widely validated 12-item instrument, measured teacher self-efficacy (Πούλου, 2007; Klassen and Chiu, 2008). This scale assesses efficacy across three domains: student engagement, instructional strategies, and classroom management. Participants rated their perceived capability on a 9-point scale from 1 (Nothing) to 9 (A Great Deal). The scale has demonstrated reliability in various international contexts. Additionally, the Instructional Confidence and Institutional Support Scale, consisting of 10 items, was created to evaluate two constructs. Five items assessed instructional confidence related to GenAI (e.g., “I am confident in my ability to critically evaluate AI-generated content for my classroom”), while the remaining five measured perceived institutional support (e.g., “My school provides adequate professional development on using GenAI effectively”), a factor shown to be crucial in technology adoption (Hazzan-Bishara et al., 2025). Responses were recorded on a 5-point Likert scale from 1 (Strongly Disagree) to 5 (Strongly Agree).

### 3.5 Data analysis

Quantitative data were analyzed using SPSS version 28. First, descriptive statistics were calculated for all demographic and primary variables. The internal consistency of all scales was assessed using Cronbach’s alpha. Pearson correlation coefficients were computed to examine the bivariate relationships between GenAI adoption, TSE, instructional confidence, institutional support, and years of experience. Finally, two hierarchical multiple regression analyses were conducted to determine the predictive power of GenAI adoption on TSE and instructional confidence, while controlling for demographic variables. A moderation analysis was performed using the PROCESS macro for SPSS to test the role of institutional support, and qualitative data, including interview transcripts, were analyzed using thematic analysis. The process involved six phases: (1) familiarization with the data through repeated reading; (2) generating initial codes from the data; (3) searching for patterns and collating codes into potential themes; (4) reviewing and refining the themes; (5) defining and naming the final themes; and (6) writing the report. Two researchers independently coded a subset of the transcripts to establish inter-rater reliability, resolving discrepancies through discussion to ensure the thematic framework’s trustworthiness. To enhance the validity of our findings, we employed methodological triangulation. This technique involved systematically comparing qualitative themes with quantitative results to identify points of convergence and divergence. For instance, the qualitative theme of “GenAI as an Efficiency Engine” was used to explain the strong statistical correlation found between GenAI use and higher self-efficacy. This approach ensures a more robust and comprehensive interpretation of the results by using one method to validate and enrich the findings of the other, thereby addressing the rigor of our qualitative analysis beyond simple inter-rater agreement.

## 4 Results

### 4.1 Quantitative findings

*Participant demographics*: The final sample for the quantitative phase included 512 K-12 teachers in Ghana. The demographic details of the participants are summarized in Table 1. The sample was diverse in terms of teaching experience, grade level, and school type, offering a broad representation of the Ghanaian K-12 teaching population.

**Table 1:** Participant demographics (*N* = 512).

Characteristic	Category	Frequency	Percentage (%)
Years of experience	0–5 years (novice)	154	30.1
	6–15 years (mid-career)	230	44.9
	16 + years (veteran)	128	25.0
Grade level	Primary (grades 1–6)	184	35.9
	Junior high (JHS 1–3)	205	40.0
	Senior high (SHS 1–3)	123	24.1
School type	Public	394	77.0
	Private	118	23.0
Region	Greater Accra	133	26.0
	Ashanti	118	23.0
	Northern	87	17.0
	Other	174	34.0

*Descriptive statistics and scale reliability*: The descriptive statistics and Cronbach’s alpha coefficients for the main study variables are presented in Table 2. All scales exhibited good to excellent internal consistency, with alpha values ranging from 0.85 to 0.94. On average, Ghanaian teachers reported moderate use of GenAI (*M* = 3.15), accompanied by high levels of teacher self-efficacy (*M* = 7.58) and instructional confidence (*M* = 3.89). Perceived institutional support was moderate (*M* = 3.05), indicating a mixed experience with school-level support for GenAI integration.

**Table 2:** Descriptive statistics and reliability of scales.

Variable	*M*	SD	Cronbach’s *α*
Overall GenAI adoption	3.15	0.98	0.88
Teacher self-efficacy (TSE)	7.58	1.12	0.94
Instructional confidence	3.89	0.85	0.91
Institutional support	3.05	1.05	0.85

*Frequency of GenAI use*: Table 3 illustrates the frequency with which teachers report using GenAI for specific instructional tasks. The most common uses were for lesson planning and creating instructional materials, which makes sense given the need for teachers to develop resources in situations where they might be limited. Use for assessment and communication was less frequent. This indicates that teachers mainly use GenAI for preparation and content creation tasks.

**Table 3:** Frequency of GenAI use for different instructional tasks (scale 1–5).

Task	*M*	SD
Lesson planning	3.85	1.10
Creating instructional materials	3.79	1.15
Differentiating instruction	3.10	1.21
Generating assessments	2.88	1.25
Communication	2.55	1.18
Administrative tasks	2.73	1.20

*Correlations*: Pearson correlation analysis (Table 4) revealed significant positive relationships among the key variables. Overall GenAI adoption was moderately and positively correlated with both TSE (*r* = 0.42, *p* < 0.001) and instructional confidence (*r* = 0.48, *p* < 0.001). Instructional confidence and TSE were also strongly correlated (*r* = 0.65, *p* < 0.001). Institutional support showed significant positive correlations with GenAI adoption, TSE, and instructional confidence. Years of experience had a small, non-significant negative correlation with GenAI adoption, indicating that veteran teachers were slightly less likely to adopt these tools. However, the effect was not statistically significant.

**Table 4:** Pearson correlations among key variables.

Variable	1	2	3	4	5
1. GenAI adoption	—				
2. TSE	0.42**	—			
3. Instructional confidence	0.48**	0.65**	—		
4. Institutional support	0.35**	0.31**	0.41**	—	
5. Years of experience	−0.07	0.11*	0.09	0.05	—

*p < 0.05, *p < 0.01.

*Regression analyses*: Two hierarchical multiple regression models were built to predict TSE and instructional confidence. For TSE (Table 5), after accounting for years of experience and school type in Step 1, the specific types of GenAI use were added in Step 2. The final model was significant (*F*(8, 503) = 25.81, *p* < 0.001) and explained 29.1% of the variance in TSE (Δ*R*^2^ = 0.27). GenAI use for lesson planning (*β* = 0.24, *p* < 0.001) and differentiating instruction (*β* = 0.21, *p* < 0.001) were identified as the strongest significant predictors.

**Table 5:** Hierarchical regression predicting teacher self-efficacy (TSE).

Predictor	*B*	SE	*β*
Step 1 (*R*^2^ = 0.02)			
Years of experience	0.05	0.02	0.11*
School type (public = 0, private = 1)	0.18	0.09	0.09
Step 2 (Δ*R*^2^ = 0.27)			
Lesson planning	0.25	0.05	0.24***
Material creation	0.08	0.05	0.08
Differentiation	0.19	0.04	0.21***
Assessment generation	0.04	0.04	0.05
Communication	−0.02	0.04	−0.02
Administrative tasks	0.06	0.04	0.07

*p < 0.05, ***p < 0.001.

For instructional confidence (Table 6), the final model was also significant (*F*(8, 503) = 35.14, *p* < 0.001) and explained 35.8% of the variance (Δ*R*^2^ = 0.34). Usage of GenAI for lesson planning (*β* = 0.28, *p* < 0.001), material creation (*β* = 0.15, *p* < 0.01), and differentiation (*β* = 0.19, *p* < 0.001) were all significant positive predictors.

**Table 6:** Hierarchical regression predicting instructional confidence.

Predictor	*B*	SE	*β*
Step 1 (*R*^2^ = 0.02)			
Years of experience	0.04	0.02	0.09
School type (public = 0, private = 1)	0.15	0.08	0.08
Step 2 (Δ*R*^2^ = 0.34)			
Lesson planning	0.23	0.04	0.28***
Material creation	0.12	0.04	0.15**
Differentiation	0.13	0.03	0.19***
Assessment generation	0.05	0.03	0.07
Communication	0.01	0.03	0.01
Administrative tasks	0.03	0.03	0.04

**p < 0.01, ***p < 0.001.

*Moderation analysis*: The moderation analysis showed a significant interaction effect between overall GenAI adoption and institutional support in predicting instructional confidence (*B* = 0.12, SE = 0.04, *p* < 0.01). This suggests that the positive link between GenAI use and instructional confidence is stronger for teachers who perceive higher levels of institutional support. For teachers with low institutional support, the impact of GenAI adoption on confidence was positive but modest, while for those with high support, the impact was much stronger.

### 4.2 Qualitative findings

Thematic analysis of 20 teacher interviews identified four major themes that clarify and add depth to the quantitative findings. These themes reveal the subtle ways in which GenAI adoption influences teachers’ professional lives and beliefs within the Ghanaian context.

Theme 1*: GenAI as an “Efficiency Engine” Nearly all participants described GenAI as a powerful tool for automating time-consuming tasks, freeing up mental and time resources for more meaningful activities. This was especially appreciated given the large class sizes and heavy administrative duties. This efficiency boost was a key factor in increasing their confidence. “My JHS class has 65 students. Before, writing report card comments would take me a whole weekend. Now, I give ChatGPT the key points for each student, and it generates a draft. I edit it, of course, but it saves me hours. This extra time I now have allows me to actually talk to the students who are struggling. It makes me feel like a better teacher, not just a record-keeper.*

Theme 2: *GenAI as a “Creative Catalyst” Teachers reported using GenAI not just to automate but to enhance their creativity. They described it as a “thought partner” that helped them develop more engaging lessons aligned with the NaCCA curriculum, even with limited resources.* “We are supposed to teach using inquiry-based methods, but sometimes it is hard to think of good activities. I was teaching about local ecosystems. I asked the AI for ideas for a low-cost project. It suggested a ‘Bio-Blitz’ where students document plants and insects around the school compound using their phones. The students loved it. I feel my teaching is more innovative now, more aligned with the new curriculum.” *(Primary Science Teacher, Kumasi, 15 years’ experience).*

Theme 3: *The “Differentiation Dilemma” While GenAI was praised for its potential to help differentiate instruction in mixed-ability classrooms, teachers also faced a major challenge: ensuring the quality and relevance of AI-generated content. This created a tension between the tool’s possibilities and the professional duty to review materials.* “It’s amazing for creating simpler texts for my weaker readers. However, you cannot just copy and paste. Sometimes the examples it gives are American or British. I have to change ‘sidewalk’ to ‘pavement’ or ‘zucchini’ to something we actually eat here. So, my confidence to differentiate is higher, but it comes with this new task of being a cultural and critical editor. You must understand your content and your students better than ever.” *(JHS Social Studies Teacher, Tamale, 5 years’ experience).*

Theme 4: *A “Confidence Paradox” This theme highlights the double-edged nature of GenAI adoption. While teachers felt more capable in many areas, they also experienced new concerns about ethics, academic honesty, and the digital divide. This paradox was especially clear in schools with little institutional guidance from the GES or headmasters.* “On one hand, I feel like a super-teacher with this tool. I can create amazing things for my students. On the other hand, I am constantly worried. Are my students using smartphones to cheat on their homework? What about the students who have no internet at home? Is this making the gap wider? My headmaster hasn’t said anything about it, so we are all just guessing. It is empowering and terrifying at the same time.” *(SHS Integrated Science Teacher, Cape Coast, 12 years’ experience).*

## 5 Discussion

This study aimed to extend the understanding of GenAI adoption beyond its antecedents to examine its impact on teachers’ self-efficacy and instructional confidence in Ghana. The mixed-methods findings provide a compelling and nuanced picture, suggesting that GenAI, when used effectively, can serve as a significant source of professional empowerment for educators in a developing country context. The quantitative results established a clear, positive relationship between the frequency of GenAI use and teachers’ efficacy beliefs. This finding extends previous research, which has essentially positioned self-efficacy as a predictor of technology adoption (Hazzan-Bishara et al., 2025; Mirriahi et al., 2025), by demonstrating a potential reciprocal effect. The act of successfully using GenAI for core teaching tasks appears to function as a powerful “mastery experience,” which, according to Bandura’s Social Cognitive Theory, is the most influential source of self-efficacy (Πούλου, 2007). The qualitative theme of “The Efficiency Engine” directly supports this; by reducing workload and cognitive load on routine tasks (Walter, 2024), particularly in the context of large Ghanaian classrooms, GenAI allows teachers to allocate their efforts to more complex pedagogical challenges, leading to more frequent experiences of success and, consequently, higher efficacy.

Furthermore, the regression analyses highlighted that the impact of GenAI is not uniform across all tasks. Its use for high-leverage activities, such as lesson planning and differentiation, was most predictive of increased efficacy and confidence. This is explained by the “Creative Catalyst” theme from our interviews. Teachers in Ghana are not merely offloading work; they are engaging with GenAI as a collaborative partner to enhance their pedagogical design, often to overcome resource limitations (Butler-Ulrich et al., 2024). This process of co-creation enhances their creative and instructional capabilities, directly boosting their confidence in designing effective and engaging learning experiences aligned with national curriculum standards. The findings from Ghana align with a growing body of international research while highlighting context-specific nuances. The “Efficiency Engine” theme, for instance, is a globally recognized benefit of GenAI in organizational settings (Agrawal, 2023). However, its impact is likely magnified in Ghana, where teachers often face larger class sizes and less administrative support than their counterparts in many countries in the Global North. Similarly, the “Creative Catalyst” function is widely discussed (Fox, 2025), but in Ghana, it plays a critical role in bridging resource gaps, a challenge less pronounced in well-funded educational systems.

The “Differentiation Dilemma” theme provides crucial context to these positive findings, especially in Ghana’s diverse classrooms. While teachers feel more confident in their ability to cater to diverse learners, this confidence is contingent on their own professional judgment and cultural awareness. They recognize the need for critical evaluation and adaptation of AI-generated content, highlighting that GenAI is a tool to augment, not replace, their pedagogical expertise (Asrori and Setyaningsih, 2025). This underscores the importance of what some have termed “AI literacy” for teachers a competency that extends beyond technical skills to encompass critical and ethical evaluation (Chen, 2025). This dilemma reflects broader concerns about the accuracy and reliability of GenAI outputs noted in other studies (Chan, 2023). This highlights the significance of what is often called “AI literacy” for teachers, a skill set that goes beyond technical knowledge to include critical thinking and ethical considerations (Le et al., 2026; Acosta, 2026). This issue also mirrors wider international worries about the accuracy and dependability of GenAI outputs, as discussed in other research (Kumar et al., 2026).

The significant moderating role of institutional support is a key finding with direct practical implications for the Ghanaian education system. The positive impact of GenAI on instructional confidence is amplified in environments where teachers feel supported through training, resources, and clear policies from their headmasters and the GES. This aligns with studies that identify institutional support as a critical enabler of technology adoption (Hazzan-Bishara et al., 2025). The “Confidence Paradox” theme further illuminates this point: without institutional guidance on ethical use and academic integrity, the empowerment offered by GenAI can be overshadowed by anxiety and uncertainty about the widening digital divide (Wang et al., 2024). This duality mirrors the characterization of GenAI as both a ‘friend’ and a ‘foe’ in the educational sphere (Lim, 2023). This duality mirrors the characterization of GenAI as both a ‘friend’ and a ‘foe’ in the educational sphere and reflects a global challenge. A worldwide review of university policies found that while institutions are proactively addressing academic integrity, significant gaps remain in developing comprehensive frameworks, especially regarding data privacy and equity (Jin et al., 2024). The anxieties expressed by Ghanaian teachers are thus not isolated but part of a larger, international conversation about responsible AI integration (Rana et al., 2024).

### 5.1 Implications for practice

The findings of this study offer several actionable implications for stakeholders in the Ghanaian education system. The Ghana Education Service and teacher training institutions should design professional development that evolves beyond introductory “how-to” sessions. Training must focus on strategic pedagogical integration, advanced prompt engineering for local contexts, and developing teachers’ critical evaluation skills to vet and adapt AI-generated content for cultural and curricular relevance. This will help teachers move from simple users to sophisticated co-creators with AI (Walter, 2024). School leaders and the Ministry of Education must create a supportive ecosystem. This includes providing access to reliable GenAI tools where possible, but more importantly, establishing transparent and fair usage policies regarding academic integrity and the ethical use of these tools. Fostering professional learning communities where teachers can share best practices and navigate challenges collaboratively is paramount (Michel-Villarreal, 2023). Our findings show that such support directly enhances the confidence-building potential of these tools, a factor also identified as crucial for leader efficacy (Leithwood and Jantzi, 2008). Pre-service teacher training colleges in Ghana must incorporate AI literacy as a core competency. Future educators need to enter the profession not only with knowledge of these tools but also with a framework for using them ethically, effectively, and critically to enhance their instructional practices, considering the realities of the digital divide in Ghana.

### 5.2 Limitations and future research

This study has several limitations. First, the cross-sectional nature of our quantitative data precludes causal claims. Although we found a strong positive association between GenAI use and self-efficacy, we cannot definitively conclude that adoption increases efficacy. It is equally plausible that teachers with pre-existing higher confidence and digital literacy are more inclined to adopt and experiment with new technologies. The relationship is likely reciprocal, with initial confidence driving adoption and successful use, in turn, reinforcing that confidence. The data are also based on self-report measures, which may be subject to social desirability bias (Eva and Regehr, 2005). Although the sample was drawn from multiple regions in Ghana, it was not a random national sample, limiting generalizability to the entire country. Furthermore, our model did not account for all potential confounding variables. Factors such as teachers’ general digital literacy, the quality of their pre-service pedagogical training, and specific school-level infrastructure could also explain variations in instructional confidence and may mediate the relationship we observed. Finally, the study focused on teachers who already had some access to and experience with GenAI, potentially excluding the perspectives of teachers on the other side of the digital divide.

To overcome these limitations, future research should employ more robust designs. Longitudinal studies are essential to track changes in teachers’ efficacy and confidence over time as they integrate GenAI into their practice, thereby providing stronger evidence of a directional effect. Moreover, experimental or quasi-experimental research could more rigorously test the causal impact of specific GenAI-based professional development interventions on teacher competence and student outcomes. Such studies would help determine whether the observed effects are genuinely transformative or primarily perceptual and context-dependent, thereby validating the findings of this exploratory study. Further investigation is needed to compare teachers’ experiences in public and private schools, and in urban and rural settings, more systematically. Finally, exploring the specific needs and adoption patterns of teachers in different subject areas, such as Twi language instruction or vocational education, would be a valuable direction (Krismiyati et al., 2026).

## 6 Conclusion

The integration of Generative AI into education is not merely a technological update; it is a phenomenon that is actively reshaping the professional lives and beliefs of teachers in Ghana. This study provides robust mixed-methods evidence that the adoption of GenAI tools is significantly and positively associated with increases in teacher self-efficacy and instructional confidence. By functioning as an “efficiency engine” and a “creative catalyst,” GenAI empowers teachers, providing them with mastery experiences that enhance their sense of professional competence, even in resource-constrained environments. However, this positive potential is not unconditional. The presence of strong institutional support moderates it and must be navigated with a critical awareness of the technology’s limitations, contextual relevance, and ethical complexities. The “confidence paradox” revealed in our findings serves as a crucial reminder that empowerment and anxiety can coexist in this new landscape, particularly concerning issues of equity. To fully realize the benefits of GenAI, the Ghanaian educational system must invest in comprehensive, context-aware training, create supportive policies, and foster a culture of critical and ethical use. By doing so, we can ensure that Generative AI becomes a tool that truly augments the irreplaceable expertise of human teachers (Chen, 2025), empowering them to meet the challenges of 21st-century education with greater confidence and capability.

## Data Availability

The original contributions presented in the study are included in the article/supplementary material, further inquiries can be directed to the corresponding author.
